# Indigenous Health and Socioeconomic Status in India

**DOI:** 10.1371/journal.pmed.0030421

**Published:** 2006-10-24

**Authors:** S. V Subramanian, George Davey Smith, Malavika Subramanyam

**Affiliations:** 1 Harvard School of Public Health, Boston, Massachusetts, United States of America; 2 University of Bristol, Bristol, United Kingdom; University of Otago, New Zealand

## Abstract

**Background:**

Systematic evidence on the patterns of health deprivation among indigenous peoples remains scant in developing countries. We investigate the inequalities in mortality and substance use between indigenous and non-indigenous, and within indigenous, groups in India, with an aim to establishing the relative contribution of socioeconomic status in generating health inequalities.

**Methods and Findings:**

Cross-sectional population-based data were obtained from the 1998–1999 Indian National Family Health Survey. Mortality, smoking, chewing tobacco use, and alcohol use were four separate binary outcomes in our analysis. Indigenous status in the context of India was operationalized through the Indian government category of scheduled tribes, or Adivasis, which refers to people living in tribal communities characterized by distinctive social, cultural, historical, and geographical circumstances.

Indigenous groups experience excess mortality compared to non-indigenous groups, even after adjusting for economic standard of living (odds ratio 1.22; 95% confidence interval 1.13–1.30). They are also more likely to smoke and (especially) drink alcohol, but the prevalence of chewing tobacco is not substantially different between indigenous and non-indigenous groups. There are substantial health variations within indigenous groups, such that indigenous peoples in the bottom quintile of the indigenous-peoples-specific standard of living index have an odds ratio for mortality of 1.61 (95% confidence interval 1.33–1.95) compared to indigenous peoples in the top fifth of the wealth distribution. Smoking, drinking alcohol, and chewing tobacco also show graded associations with socioeconomic status within indigenous groups.

**Conclusions:**

Socioeconomic status differentials substantially account for the health inequalities between indigenous and non-indigenous groups in India. However, a strong socioeconomic gradient in health is also evident *within* indigenous populations, reiterating the overall importance of socioeconomic status for reducing population-level health disparities, regardless of indigeneity.

## Abstract 2

Indigenous groups in India were found to have excess mortality rates compared with non-indigenous groups. A socioeconomic gradient within indigenous populations was also found.

## Editors' Summary

**Background.:**

In many parts of the world the majority of the population are the descendants of immigrants who arrived there within the last few hundred years. Living alongside of them, and in a minority, are the so-called indigenous (or aboriginal) people who are the descendants of people who lived there in more ancient times. It is estimated that there are 300 million indigenous people worldwide. They are frequently marginalized from the rest of the population, their human rights are often abused, and there are serious concerns about their health and welfare. The state of health of the indigenous people of developed countries such as the US and Australia has often been studied, and we have a fairly clear idea of the kinds of problems these people face. Most indigenous people, however, live in developing countries, and less is known about their health.

India is the second-most populous country in the world, with an estimated 1.1 billion inhabitants. An estimated 90 million indigenous people live in India, where they are often referred to as “scheduled tribes” or Adivasis. They live in many parts of the country but are much more numerous in some Indian states than in others.

**Why Was This Study Done?:**

It has often been said that indigenous people in India have worse health than other Indians, though no figures have been compiled to confirm these claims. The researchers wanted to establish whether it is simply an issue of indigenous people being poorer than other Indians—poverty being well known as a cause of disease—or whether being indigenous is, in itself, a health risk. The researchers also wanted to establish whether there are health inequalities within indigenous groups, and if these differences also followed a socioeconomic patterning.

**What Did the Researchers Do and Find?:**

They used figures collected in the 1998–1999 Indian National Family Health Survey. When this survey was conducted, it was noted whether people were considered to be members of scheduled tribes. The researchers also knew, from the survey, about the income of the families, their death rates, and whether they drank alcohol or smoked or chewed tobacco. They found that indigenous people had higher death rates than other Indians. They made statistical calculations to account for differences in standard of living, and this substantially reduced the difference in death rate among indigenous groups, but an indigenous person was still 1.2 times more likely to die than a non-indigenous person with the same standard of living. Indigenous people were also more likely to drink alcohol and smoke tobacco, and here again, differences in standard of living accounted for a substantial portion of the differences. Importantly, the researchers' analysis showed a strong socioeconomic patterning of health inequalities within the indigenous population groups: the health differences between the poorest and richest indigenous groups were similar in scale to the differences between the poorest and richest non-indigenous groups.

**What Do These Findings Mean?:**

The authors consider their finding that there is a socioeconomic gradient in mortality and health behaviors among indigenous people to be an important result from the study. The socioeconomic marginalization of indigenous people from the rest of Indian society does seem to increase their health risks, and so does their use of alcohol and tobacco. However, if their standard of living can be improved there would be major benefits for their health and welfare.

**Additional Information.:**

Please access these Web sites via the online version of this summary at http://dx.doi.org/10.1371/journal.pmed.0030421.

A useful discussion of the term “indigenous people” (with links to documents about international agreements intended to improve their human rights) may be found on Wikipedia. (Wikipedia is an internet encyclopedia that anyone can edit.)

- Survival International is a human rights organization that campaigns for the rights of indigenous peoples, helping them preserve their land and culture.
- The charity Health Unlimited also works with indigenous people and its Web site includes links to recent studies and conferences.
- A news item from the BBC describes a recent investigation into the health of indigenous people worldwide.
- The World Health Organization has produced a number of reports on the health of indigenous people

## Introduction

Indigenous people are amongst the poorest and most marginalized population groups experiencing extreme levels of health deprivation [1]. The suboptimal health status of indigenous peoples and the health inequalities between indigenous and non-indigenous populations reflect a fundamental failure to ensure the freedom of indigenous peoples to fully realize their human, social, economic, and political capabilities [2]. Importantly, the health and wealth disparities between indigenous and non-indigenous populations are universal [3,4]. Improving indigenous health as well as eliminating the indigenous/non-indigenous health divide requires addressing the knowledge gap related to understanding the patterns of indigenous health deprivation [3]. Surveillance of, as well as research on, indigenous health remains inadequate [3], even though this gap is beginning to be bridged in developed countries [5–7]. While the unfavorable health status of indigenous peoples in developed countries has been shown across a range of outcomes, including mortality [8], disease [9], health behaviors [10,11], and health care [12,13], there are few systematic accounts of the health of indigenous peoples in developing countries [14,15].

This study examines the patterns of health deprivation amongst indigenous populations in India. Notwithstanding the challenges of defining indigenous populations [16], including those specific to India [17,18], the group classified by the Indian government as “scheduled tribes” has often been categorized as being indigenous [19,20]. Over 84 million people belonging to 698 communities are identified as members of scheduled tribes [18], constituting 8.2% of the total Indian population [21]. Through a constitutional mandate [18], formulated in 1950, scheduled tribes have been formally recognized as a distinct community in India. Consequently, there exist clear governmental policies for affirmative actions targeted towards scheduled tribes [22], and their members are routinely enumerated in national surveys [23] and censuses [21]. The Indian government identifies communities as scheduled tribes based on a community's “primitive traits, distinctive culture, shyness with the public at large, geographical isolation and social and economic backwardness” [18], with substantial variations in each of these dimensions with respect to different scheduled tribe communities [24]. While “scheduled tribes” is an administrative term adopted by the Government of India, the term “Adivasis” (meaning “original inhabitants” in Sanskrit) is often used to describe the different communities that belong to scheduled tribes. The Adivasis are thought to be the earliest settlers in, and the original inhabitants of, the Indian peninsula, with their presence dating back to before the Aryan colonization (pp. 37–38 of [25]). The distinct identity of Adivasis has many aspects: language, religion, a profound bond linking the individual to the community and to nature, minimal dependence on money and markets, a tradition of community-level self-government, and an egalitarian culture that rejects the rigid social hierarchy of the Hindu caste system [26], all of which closely approximates the indigenous definition articulated at the international level [27]. Since the formal recognition of scheduled tribes in 1950, the proportion of individuals of scheduled tribes in the total Indian population has increased from 5.3% (1951) to 8.2% (2001) [18]. The concentration of scheduled tribes varies substantially between the Indian states [21]. In northeastern states, scheduled tribes constitute 65% or more of the total population; in Chattisgarh, Jharkhand, Orissa, Madhya Pradesh, Gujarat, and Rajasthan this proportion ranges between 13% and 32% of the population; and in other states, including Punjab, Haryana, Delhi, and Goa, the contribution of scheduled tribes to the total population is negligible. In this study, we consider the scheduled tribe category as being equivalent to indigenous within the Indian context.

Existing research on indigenous health in India, as in many developing countries [28–30], is restricted to specific indigenous groups [31,32]. The ability to meaningfully generalize the extent and nature of indigenous health patterns in India, consequently, remains limited. Using a nationally representative sample, we investigate the extent to which the indigenous/non-indigenous health divide is a reflection of the differences in socioeconomic well-being between indigenous and non-indigenous groups. If differential distribution of socioeconomic resources accounts for indigenous/non-indigenous health inequalities, this would emphasize the need to redress the pervasive and chronic socioeconomic inequalities between the indigenous and non-indigenous groups. Furthermore, we also examine the extent to which socioeconomic well-being predicts health outcomes *within* indigenous populations. If health inequalities are fundamentally social in nature [33,34], and have less to do with being indigenous, we should expect a socioeconomic gradient in health even within this marginalized population. The patterns of indigenous health deprivation and heterogeneity are investigated for all-cause mortality and tobacco and alcohol consumption; the public health relevance of tobacco and alcohol use in India has been well documented in recent years [35–43].

## Methods

### Data

The analyses are based on the representative cross-sectional 1998–1999 Indian National Family Health Survey (INFHS) household data [23]. The household data were obtained by face-to-face interviews, conducted in one of the 18 Indian languages, in the respondent's own home, and information was obtained on a range of health, demographic, and socioeconomic topics for each member of the household. The survey response rate ranged from 89% to almost 100%, with 24 of the 26 states having a rate of more than 94% [23]. All households were geocoded to the primary sampling unit, district, and state to which they belonged. The primary sampling units, hereafter called “local areas,” were villages or groups of villages in rural areas, and wards or municipal localities in urban areas.

### Outcomes

The study analyzed the health inequalities between indigenous and non-indigenous groups across four different outcomes: mortality, smoking tobacco use, chewing tobacco use, and alcohol consumption. All four outcomes were measured at the level of individuals and were analyzed separately; the lowest unit of observation for this study was the individual. We briefly describe the survey-based definition of each of the outcome variables.

#### Mortality.

The respondent to the household survey (typically the head of the household) was asked about the number of living resident members of the household and the number who had died in the 2 y (1997–1998) preceding the survey. For each deceased household member, information was obtained on gender and age at death. The total number of household members who were alive at the time of the survey was 517,379, and the number of deaths reported for the previous 2 y was 11,827. For the mortality analysis, we thus observed a sample of 529,206 individuals (number of household members alive and dead). This constituted the basis for defining the outcome variable mortality, which was then modeled as a dichotomous outcome (one if an individual was dead, zero if alive) [23]. Thus, mortality was an outcome even though it was estimated from a cross-sectional survey. Such indirect methods of mortality assessment are widely utilized in demographic studies, and their suitability for this is widely tested [44,45].

#### Smoking behavior, tobacco chewing, and alcohol use.

The respondent to the household survey was also asked, via three separate questions, “Does anyone listed as a member of this household in this survey smoke/chew tobacco/drink alcohol?”

### Independent Variables

Predictors were measured at the individual and at the household level simultaneously. For the mortality analysis, the individual predictor variables that were common to both the deceased and alive household members were gender and age. At the household level, the respondent to the household survey was asked about (1) whether he or she was a member of a scheduled tribe (our operational definition of indigenous), (2) religious affiliation, and (3) the possession of various production and consumption assets. Under the reasonable assumption that characteristics associated with being indigenous, religious affiliation, and standard of living would not have changed in the 2-y window when mortality was reported, we assigned the values of the household predictors to both the deceased and alive household members. Thus, for the mortality analysis, the predictors included age and gender at the individual level, and ethnicity, religion, standard of living, and urban/rural at the household level.

Standard of living—the key indicator for socioeconomic status common to the analysis pertaining to mortality and the three health behaviors—was measured by household assets and material possessions. While there is some argument about the relative merits of using asset, consumption, or income data to measure economic well-being, empirical evidence suggests that there is a strong positive association between the three types of data, and as such an asset index, as used here, is a reliable proxy for household income [46,47]. To the extent this is true, asset ownership can be considered a reasonable proxy for consumption, in addition to being an indicator of economic status in its own right, and this has also been validated in the Indian context [48]. We adapted the INFHS standard of living index to the “proportionate possession weighting” used in studies of poverty in a number of countries [49–51]. The INFHS standard of living index and the weighted standard of living index that we used were correlated to the order of 0.93 (*p* < 0.001). The weights for each item were derived on the basis of the proportion of households owning the particular item. Thus, for example, if 40 of the households in a sample of 100 owned a radio, then a radio would get a weight of 60 (100 − 40). Weights for each item were summed into a linear index, and households were allocated a final score. Since the standard of living index is a constructed composite measure, it does not have a direct interpretation. We followed the convention of dividing the population into quintiles of the standard of living index for our analysis.

Urban/rural status was categorized in terms of the location of the household: large city (population ≥ 1 million), small city (population 100,000–1 million), town (population ≤ 100,000), or village/rural area.

For the analysis related to tobacco and alcohol consumption, we additionally could specify marital status and educational attainment (in terms of years of schooling) at the individual level. Tables 1 and 2 present the prevalence of mortality (Table 1) and tobacco and alcohol use (Table 2) in the sample population disaggregated for indigenous and non-indigenous populations by the different variables studied.

**Table 1:**
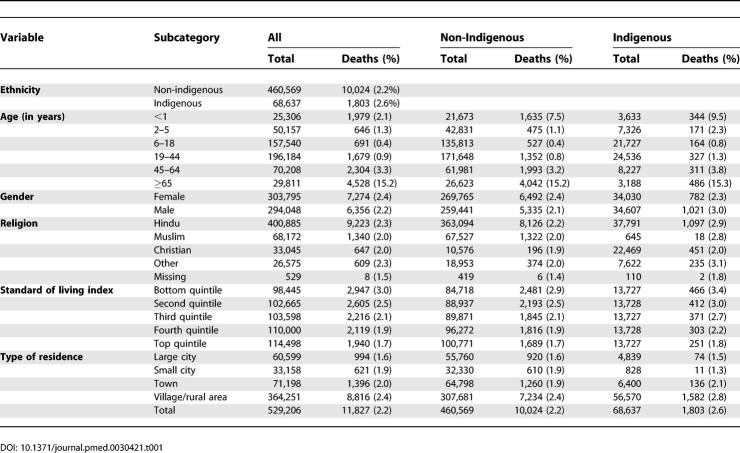
Descriptive Information on the Analytic Sample Considered for the Mortality Analysis (1998–1999 INFHS)

**Table 2:**
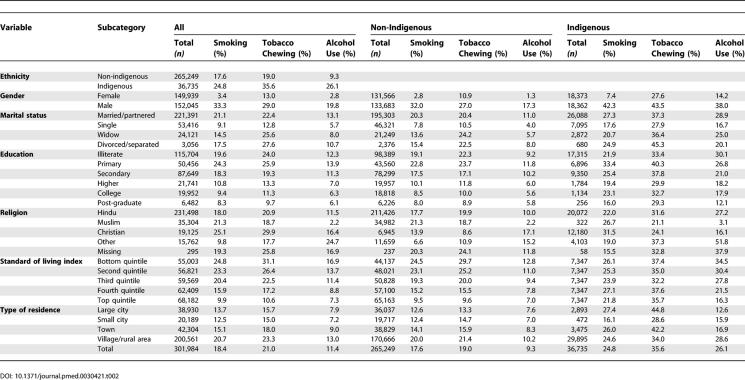
Descriptive Information on the Analytic Sample Considered for the Health Behavior Analyses (1998–1999 INFHS)

### Statistical Analysis

We modeled the variation in mortality and tobacco and alcohol use using a multilevel modeling approach [52]. The binary response, *y* (dead or not; smoke or not; chew tobacco or not; drink alcohol or not), for individual *i* living in local area *j* in district *k* in state *l* was formulated as:

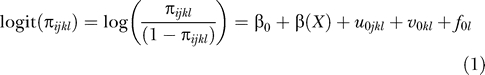



The equation consists of a fixed part, β_0_ + β(*X*), and random effects attributable to local areas (*u*_0*jkl*_), districts (*v*_0*kl*_), and states (*f*_0*l*_). The parameter β_0_ estimates the log odds in the outcome for the reference group, and the parameters β(*X*) estimate the differential in the log odds in the outcome for the different predictors. Assuming an independent and identical distribution, the random effects are summarized as 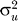 (local areas), 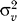 (districts), and 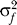 (states). These variance parameters quantify the heterogeneity in the outcome at each level, thus being suggestive of the independent importance of geographic contexts [53]. Model estimates are marginal quasi-likelihood-based with a first-order Taylor linearization procedure [52,54]. Models were stratified for indigenous and non-indigenous samples, and a formal test of interaction was conducted to test for the differentials by the same predictor in the two populations [55].

Specifically, we calibrated the following types of models: (1) a pooled model of all indigenous and non-indigenous samples separately for mortality, smoking, drinking alcohol, and chewing tobacco (Table 3); (2) a gender-stratified model of all indigenous and non-indigenous samples separately for mortality, smoking, drinking alcohol, and chewing tobacco (Table 3); (3) an age-stratified model of all indigenous and non-indigenous samples for mortality (Figure 1); (4) a stratified model of mortality for indigenous and non-indigenous samples (Table 4); and (5) a stratified model of alcohol and tobacco use for indigenous and non-indigenous samples (Table 5).

**Table 3:**
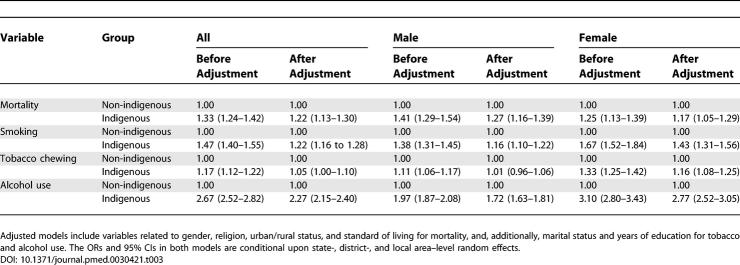
Unadjusted and Adjusted Indigenous and Non-Indigenous Differentials in Mortality and Tobacco and Alcohol Use

**Figure 1:**
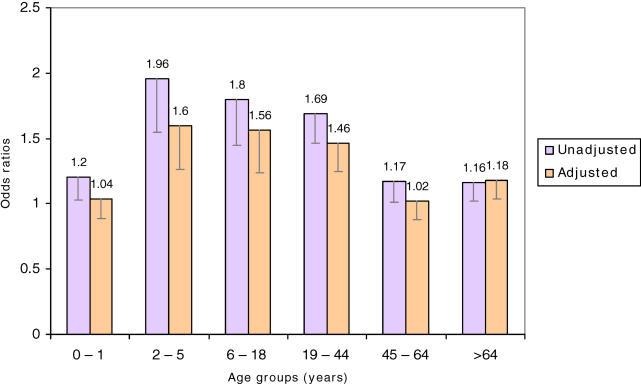
Unadjusted and Adjusted Indigenous and Non-Indigenous Differentials in Mortality across Life Stages Adjusted models include variables related to gender, religion, urban/rural status, and standard of living. The ORs and 95% CIs in both models are conditional upon state-, district-, and local area–level random effects.

**Table 4:**
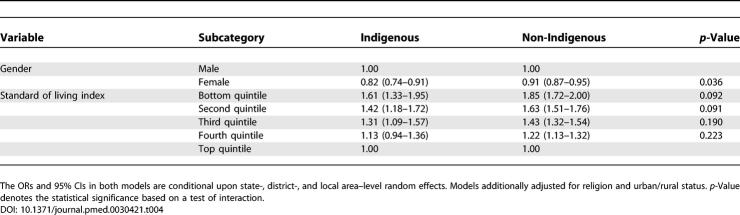
Gender and Socioeconomic Differentials in Mortality within Indigenous and Non-Indigenous Groups

**Table 5:**
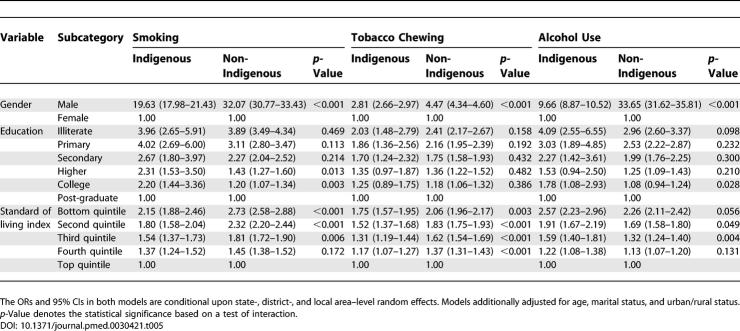
Gender and Socioeconomic Differentials in Tobacco and Alcohol Use within Indigenous and Non-Indigenous Groups

## Results

### Differentials between Indigenous and Non-Indigenous Groups

In the indigenous sample, the proportion of the total number of deaths in the total sample of individuals (alive and dead) over the 2-y period was 2.6%, compared to 2.2% in the non-indigenous sample (Table 1). Table 3 presents differentials in mortality and health behaviors between indigenous and non-indigenous populations, before and after adjusting for indicators of socioeconomic circumstances. Indigenous peoples have higher mortality (odds ratio [OR] 1.33; 95% confidence interval [CI] 1.24–1.42) than non-indigenous peoples. Adjusting for the standard of living index attenuates the OR to 1.22 (95% CI 1.13–1.30). The mortality OR for indigenous men was 1.41 (95% CI 1.29–1.54), while for indigenous women it was 1.25 (95% CI 1.13–1.39), suggesting that the indigenous/non-indigenous divide was larger for men (*p* = 0.04). This gender difference remained with adjustment for standard of living, although with weaker statistical support (*p* = 0.11).

Based on the response to the three questions, the proportions of individuals smoking, chewing tobacco, and using alcohol in the sample of indigenous populations were 25%, 36%, and 26%, respectively, compared to 18%, 19%, and 9% for non-indigenous populations (Table 2). Indigenous/non-indigenous differentials are substantial for smoking and drinking. The OR related to being indigenous for smoking was 1.47 (95% CI 1.40–1.55) and for drinking alcohol was 2.67 (95% CI 2.52–2.82). The ORs are attenuated to 1.22 (95% CI 1.16–1.28) for smoking and 2.27 (95% CI 2.15–2.40) for drinking after adjustment for standard of living. The indigenous/non-indigenous differentials for tobacco chewing are not substantial, especially after adjustment for standard of living. Even after adjusting for standard of living, the indigenous/non-indigenous differential is greater among women, than men, for both smoking and drinking (*p* < 0.001).

Figure 1 shows the excess mortality in indigenous peoples across the different stages of the life course before and after adjusting for gender, religion, urban/rural status, and standard of living index. No statistically significant differences were observed between indigenous and non-indigenous groups for mortality in the age groups under 1 y and 45–64 y; in the remaining age groups the mortality risk for indigenous groups was consistently greater than that observed for non-indigenous groups. As shown in Figure 1, it is only after adjustment for household socioeconomic status that the mortality differentials for the age group under 1 y for indigenous groups becomes substantially attenuated. This finding suggests that while there are likely to be differences in the determinants of the infant mortality (e.g., access and availability of public health measures and health-care services) between indigenous and non-indigenous groups, such differences seem to get largely accounted by the average differences in economic well-being between indigenous and non-indigenous groups. Indeed, attenuation is observed in the indigenous mortality differentials across most age groups.

### Differentials within Indigenous and Non-Indigenous Groups

#### Mortality.

As shown in Table 4 there are substantial mortality differentials by standard of living index within indigenous groups, with the OR for mortality being 1.61 (95% CI 1.33–1.95) for those in the bottom fifth of the standard of living index compared to those in the top fifth. In non-indigenous groups, the socioeconomic differential is greater, with the bottom fifth 85% more likely to experience mortality than the top fifth. The statistical evidence for a difference in the relationship between standard of living and mortality between indigenous and non-indigenous groups, however, was not strong (Table 4). The results from the stratified analysis of mortality (Table 4) were similar to those from overall models with interaction terms specified between indigenous status and standard of living. Similarly, while we report results from a stratified analysis for each of the health behaviors, we also tested for interactions between indigenous status and socioeconomic position in pooled models, and the results were similar.

#### Smoking.

Table 5 presents the adjusted socioeconomic differentials in tobacco use in indigenous and non-indigenous populations. Indigenous men are substantially more likely to smoke than non-indigenous men. The gender differential in smoking is, however, much stronger in non-indigenous groups. Indigenous groups with no education are more likely to smoke than the most educated indigenous groups (OR 3.96; 95% CI 2.65–5.91), while those in the bottom fifth of the standard of living index are more than twice as likely to smoke than those in the top fifth (95% CI 1.88–2.46). While the educational differentials in smoking are similar in indigenous and non-indigenous populations, the standard of living differentials in smoking are marginally larger within non-indigenous groups.

#### Tobacco chewing.

Within indigenous and non-indigenous groups, there are substantial gender differences in tobacco chewing, with men being much more likely to engage in this behavior than women (Table 5). The gender differentials are, however, stronger in non-indigenous groups than in indigenous groups. Indigenous people with secondary or less education (i.e., primary or no education) are more likely to chew tobacco than those with the most education. Similarly, an increased likelihood of chewing tobacco use (OR 1.75; 95% CI 1.57–1.95) is observed for those in the bottom quintile of the standard of living index (Table 5). The pattern of socioeconomic differentials in tobacco chewing is largely similar in indigenous and non-indigenous groups, with the actual differential being somewhat greater in non-indigenous groups.

#### Alcohol use.

Men, indigenous or non-indigenous, are more likely to drink alcohol than women. As with smoking and tobacco chewing, the gender differentials in alcohol use are greater in non-indigenous groups. The odds of alcohol use are four times (95% CI 2.55–6.55) greater in indigenous populations with no education and ~ 2.5 times (95% CI 2.23–2.96) greater in indigenous populations in the bottom quintile of the standard of living index, compared to those with the most education and in the top quintile of the standard of living index, respectively (Table 5). The standard of living differentials within indigenous populations are substantially larger than those observed in non-indigenous groups, a pattern different from those observed for tobacco smoking and chewing.

## Discussion

Our analysis has two major findings related to patterns of health deprivation among indigenous peoples in India. First, there are substantial differences in mortality and tobacco and alcohol consumption between indigenous and non-indigenous peoples, with all values being disproportionately greater for indigenous peoples. The differential distribution of demographic factors as well as socioeconomic status in indigenous and non-indigenous populations accounts for a substantial portion of the health inequalities between these two groups.

The relative excess mortality among indigenous peoples is greatest for children and adults up to middle age; for adults over 45 y and for infants under 1 y the differences are relatively small. As shown in Figure 1, a substantial attenuation is observed in the indigenous mortality differentials across all age groups (and especially for infants) once we adjust for differences in household socioeconomic position. This finding favors an interpretation focused on the importance of socioeconomic status over an interpretation that views indigeneity as an intrinsic risk factor. Furthermore, the differential attenuation in the mortality gap for indigenous groups across life stages may suggest that the importance of socioeconomic status is greater at younger ages than older ones. This mirrors the magnitude of socioeconomic differentials in mortality within India, which are also greatest in young age groups [56]. This result may reflect mortality related to socio-environmental factors important for childhood mortality (such as water availability and overcrowded or inadequate housing), unequal access to health care, and the patterning of health-related behaviors, including tobacco and alcohol use.

The excess use of tobacco and alcohol among indigenous groups observed in this study is important in its own right [40,41], as well as in terms of its contribution to accounting for the excess mortality [57,58], though in this study we were unable to examine the latter directly since information on tobacco and alcohol consumption was not ascertained for the deceased household members. The excess use of tobacco and alcohol in some indigenous populations has been shown to be linked to the process of colonization [10] and increased influence of Western culture [11,14], which may be in direct conflict with indigenous models of normative social behavior. The greater indigenous/non-indigenous mortality differentials in men than women may reflect the considerably higher prevalence of tobacco and alcohol use in men than women and thus their contribution to greater mortality differentials, despite relative differences in these behaviors being greater among women than among men.

An important second finding of this study is that there are substantial heterogeneities in mortality and tobacco and alcohol consumption *within* indigenous peoples. Differential educational attainment and standard of living are major producers of health-related heterogeneities even within indigenous populations. This finding reiterates the importance of social and economic well-being in creating health differences within indigenous groups, as well as between indigenous peoples and other population groups. Whilst, in general, the socioeconomic differentials within indigenous groups are smaller than those observed in non-indigenous groups they are still substantial. The presence of such differentials draws attention to the need to consider such heterogeneities within population groups that are seen as having less favorable socioeconomic and health experiences. Indeed, sometimes the comparison of the health of such groups—whether indigenous or minority ethnic groups—with the majority population can lead to indigenous group membership (or minority ethnic group status) being taken as a straightforward proxy for adverse socioeconomic circumstances. This can draw attention away from the considerably important social differences within as well as between population groups. The socioeconomic differentials observed within indigenous groups also suggest that the indicators of socioeconomic well-being such as educational attainment and material assets have at least some common meaning within indigenous groups as well as in the non-indigenous population.

The findings of our study need to be considered alongside the following limitations. The findings related to mortality are likely to be influenced by recall bias of deaths (and of the age of the dead) that occurred in the household [59]. While this is expected to be a greater limitation for cause-specific mortality analyses, the possible under-reporting of deaths remains a concern [59], with under-reporting being possibly greater for indigenous populations. Analyses of mortality differentials based on household surveys may also raise concerns related to sample sizes [60]. Consequently, caution is necessary when drawing inferences about population-level mortality estimates (in particular adult mortality estimates) based on the data source used for this study. In support of the mortality data, however, it is noted that the crude death rates estimated from the INFHS closely approximates the crude death rates obtained from more routine sources, such as the Sample Registration System (SRS), which is a large-scale demographic survey conducted in India that has historically provided the annual estimates of birth rate, death rate, and other fertility and mortality indicators at the national and sub-national levels (http://www.censusindia.net/srs21.html; accessed on March 9, 2006). The crude death rate from INFHS (covering roughly the period of 1997–1998) was 9.7 deaths per 1000 population compared with 8.9 from the 1997 SRS [23]. This finding, contrary to the expectations, actually suggests that reporting of deaths may have been better in the INFHS than in the SRS. The INFHS age-specific death rates were also higher for most of the age groups, with one notable exception, the age group 0–4 y, where the INFHS estimate is considerably lower than the SRS estimate.

Given the above, conclusions drawn from the mortality analyses presented here should be restricted to descriptive inferences on the underlying patterning of mortality differences between and within indigenous and non-indigenous groups. Furthermore, while overall mortality patterns reflect the general underlying health burden, cause-specific analysis of mortality across indigenous and non-indigenous groups is necessary to develop specific public health and medical interventions.

Our analysis also does not cover all dimensions of socioeconomic status, and some of the apparent residual worse mortality and health-related behavior could be due to unmeasured aspects of socioeconomic position [61]. For instance, since educational levels were not ascertained for deceased individuals, we could not ascertain the contribution of educational differences to the indigenous/non-indigenous mortality divide, nor to the heterogeneities in mortality within indigenous populations. Many studies have demonstrated that incorporating additional measures of socioeconomic position into studies utilizing just one dimension leads to greater differentials being demonstrated and increased statistical explanation of variance and health outcomes [62]. Consequently, what we report in this study as the contribution of socioeconomic status in attenuating the indigenous/non-indigenous health divide and the extent of socioeconomic inequalities in health within indigenous groups are likely to be underestimates of the true contribution of socioeconomic circumstances to explaining health differentials between and within population groups [59]. It is, however, possible that the socioeconomic measures considered in this study may not have the same meaning within indigenous and non-indigenous peoples, leading to inadequate control for this determinant of health differences between population groups [61].

With regards to the analysis of tobacco and alcohol consumption, the survey data did not measure the levels of consumption, or the type of alcohol or form of tobacco smoked, as such limiting us to investigating overall prevalence. It is, however, likely that including type of tobacco or alcohol consumed would only exacerbate the observed indigenous/non-indigenous disparities in health behaviors. For instance, *bidi* smoking and drinking locally produced alcohol are likely to be more common amongst indigenous groups. Finally, since the health behaviors were reported, we are unable to ascertain the extent to which the observed disparities reflect actual behavior and the degree to which there are systematic reporting gaps.

### Indigenous Population Groups in the Indian Context

The definition of indigenous peoples put forward by the International Labor Organization in Convention 169, as well as the recently revised World Bank Policy on indigenous people, supports the application of the term “indigenous” to the scheduled tribes in India [19,20]. However, the Government of India has resisted the use of the term indigenous when referring to the scheduled tribes on the grounds that it is a practical impossibility to decide indigeneity after centuries of “migration, absorption, and differentiation” [18]. As reflected in the “National Policy on Tribals” draft [18], a formal recognition of the indigeneity of the scheduled tribes is also interpreted as contradictory to the overall spirit underlying the recognition of the tribal populations [63], which is to facilitate “assimilation” of the tribal population into the country's mainstream [18]. From a political standpoint, it has been argued that official adoption of the term “indigenous” also legitimizes the potential for secession of scheduled tribal areas from the Indian state [17]. Notwithstanding the identification challenges related to “who is indigenous,” the scheduled tribes in India approximately fit the definition by Maybury-Lewis [16], who states, “Indigenous peoples are defined as much by their relations with the state as by an intrinsic characteristic that they may possess. They are often considered to be tribal people in the sense that they belong to small-scale pre-industrial societies that live in comparative isolation and manage their own affairs without the centralized authority of a state.” We would contend that many of the same forces—historical and contemporary—that lead to adverse socioeconomic and health consequences for indigenous populations in other parts of the world [1,3–7,10,11] apply to the scheduled tribe populations of India.

### Conclusions

Our analysis presents evidence for excess mortality and tobacco and alcohol use in the indigenous populations in India. This excess mortality and tobacco and alcohol use, however, is markedly attenuated once the average differences in socioeconomic well-being between the indigenous and non-indigenous groups are taken into account. Since differences in socioeconomic well-being are unlikely to account for all the indigenous/non-indigenous differentials in health, the question remains, what does the residual difference indicate? Does it reflect unobserved confounding due to biological predispositions? Does it reflect the effect of “racism”? Recent evidence from New Zealand emphasizes the importance of discrimination in explaining the adverse health status of the Maoris [64]; research on the role of discrimination in accounting for indigenous/non-indigenous health differences in India is absent, but may provide important insights into explaining the residual differences in health status between indigenous and non-indigenous groups. The presence of socioeconomic inequalities in health even within indigenous populations is a key finding of our study. The mortality differentials by material standard of living within the indigenous populations are similar to those seen within the non-indigenous population. While there are critical issues related to political and social marginalization that are central to improving the health and wealth of indigenous populations in absolute terms (in addition to reducing the gap between indigenous and non-indigenous groups), our findings suggest that a focused approach to addressing inequalities in social and economic well-being within and between the indigenous and non-indigenous populations would contribute to reducing health inequalities in a general fashion. An effective application of such approaches is likely to lead to decreasing relevance of the indigenous aspect of the experience of scheduled tribal populations, in line with the stated objectives of the Government of India.

## Abbreviations

CI: confidence interval
INFHS: Indian National Family Health Survey
OR: odds ratio
SRS: Sample Registration System
